# Supplementary material for: Modeling, state estimation, and optimal control for the US COVID-19 outbreak
Source: Sci Rep. 2020 Jul 1;10:10711. doi: 10.1038/s41598-020-67459-8 (PMC7329889; doi:10.1038/s41598-020-67459-8)
Supplement: Supplementary file 1 — Supplementary information [file 41598_2020_67459_MOESM1_ESM.pdf]

# Modeling, state estimation, and optimal control for the US COVID-19 outbreak: Supplementary Information

**Calvin Tsay[1,+], Fernando Lejarza[1,+], Mark A. Stadtherr[1], and Michael Baldea[1,2,*]**

[1]McKetta Department of Chemical Engineering, The University of Texas at Austin, Austin, TX, USA
[2]Oden Institute for Computational Engineering and Sciences, The University of Texas at Austin, Austin, TX, USA
[*]mbaldea@che.utexas.edu
[+]these authors contributed equally to this work

A least-squares regression problem was solved for the values of the parameters in the SEAIR model (1)–(6). The estimated means of the parameters for the USA, Italy, Spain, and Germany are given below in Table S1, and the covariance matrices are given in Table S2. These covariance matrices were used to generate samples for bootstrapping.

| | USA | Italy | Spain | Germany |
|---|---|---|---|---|
| $\mu$ [days$^{-1}$] | $4.405 \times 10^{-3}$ | $10.619 \times 10^{-3}$ | $11.871 \times 10^{-3}$ | $2.402 \times 10^{-3}$ |
| $\beta$ [days$^{-1}$] | $7.467 \times 10^{-3}$ | $16.644 \times 10^{-3}$ | $40.129 \pm \times 10^{-3}$ | $46.838 \times 10^{-3}$ |
| $e_0$ [people] | $1.684 \times 10^{-1}$ | $90.035 \times 10^{-1}$ | $2.004 \times 10^{-1}$ | $1.751 \times 10^{-1}$ |

**Table S1.** Mean values of time-invariant model parameters.

| USA | | | |
|---|---|---|---|
| $\beta$ [days$^{-1}$] | $1.39 \times 10^{-7}$ | | |
| $e_0$ [people] | $4.54 \times 10^{-7}$ | $2.61 \times 10^{-6}$ | |
| $\mu$ [days$^{-1}$] | $-3.59 \times 10^{-9}$ | $-2.75 \times 10^{-8}$ | $8.59 \times 10^{-9}$ |

| Italy | | | |
|---|---|---|---|
| $\beta$ [days$^{-1}$] | $3.00 \times 10^{-9}$ | | |
| $e_0$ [people] | $-2.55 \times 10^{-8}$ | $8.09 \times 10^{-4}$ | |
| $\mu$ [days$^{-1}$] | $-2.58 \times 10^{-9}$ | $3.43 \times 10^{-6}$ | $1.79 \times 10^{-8}$ |

| Spain | | | |
|---|---|---|---|
| $\beta$ [days$^{-1}$] | $4.44 \times 10^{-8}$ | | |
| $e_0$ [people] | $1.12 \times 10^{-7}$ | $8.08 \times 10^{-7}$ | |
| $\mu$ [days$^{-1}$] | $-8.89 \times 10^{-9}$ | $9.17 \times 10^{-8}$ | $3.43 \times 10^{-8}$ |

| Germany | | | |
|---|---|---|---|
| $\beta$ [days$^{-1}$] | $6.36 \times 10^{-6}$ | | |
| $e_0$ [people] | $1.47 \times 10^{-5}$ | $5.49 \times 10^{-5}$ | |
| $\mu$ [days$^{-1}$] | $5.47 \times 10^{-8}$ | $-1.45 \times 10^{-7}$ | $4.66 \times 10^{-9}$ |

**Table S2.** Covariance matrices of the time-invariant model parameters.

Given the parameters for the US outbreak, the optimization problem (8) can be solved for the next 100 days. Fig 4 shows the solutions for peak infected population $i_{\text{peak}}$ values of 700,000 and 1,400,000 people. Here, we solve (8) for various values

of $i_{\text{peak}}$ to investigate the relationship between $i_{\text{peak}}$ and the associated socioeconomic cost $C$. The results are given in Fig S1. As the cost function is in arbitrary units, we report the cost here as the percentage increase over the cost function value for normal societal operation, i.e., with no mitigation/testing strategies ($\alpha_a = 0.5$, $\alpha_i = 0.3$, $\kappa = 0.1$).

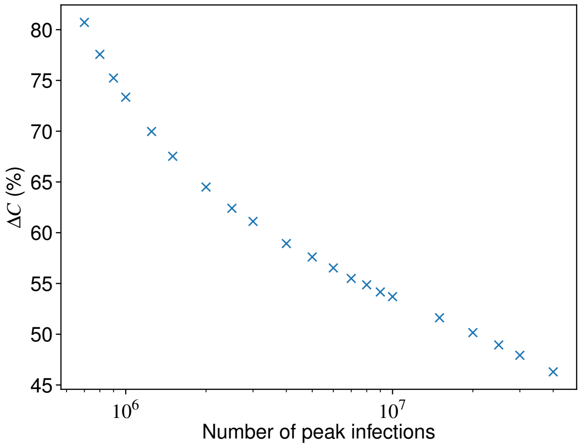


| $i_{\text{peak}}$ | $\Delta C$ (%) |
|---|---|
| $4 \times 10^7$ | 46.29 |
| $3 \times 10^7$ | 47.92 |
| $2.5 \times 10^7$ | 48.94 |
| $2 \times 10^7$ | 50.16 |
| $1.5 \times 10^7$ | 51.62 |
| $1 \times 10^7$ | 53.70 |
| $9 \times 10^6$ | 54.16 |
| $8 \times 10^6$ | 54.87 |
| $7 \times 10^6$ | 55.50 |
| $6 \times 10^6$ | 56.53 |
| $5 \times 10^6$ | 57.61 |
| $4 \times 10^6$ | 58.93 |
| $3 \times 10^6$ | 61.11 |
| $2.5 \times 10^6$ | 62.41 |
| $2 \times 10^6$ | 64.50 |
| $1.5 \times 10^6$ | 67.53 |
| $1.25 \times 10^6$ | 69.97 |
| $1 \times 10^6$ | 73.35 |
| $9 \times 10^5$ | 75.24 |
| $8 \times 10^5$ | 77.57 |
| $7 \times 10^5$ | 80.71 |

**Figure S1.** Cost required to limit peak infections to various values. Costs given as percentage increases from no-action-taken scenario.

The moving horizon strategy shown in Fig 6 involves updating control policies in 25-day intervals (after day 85). The problem solved at each update is identical, except that the initial conditions are updated based on the current measured/estimated populations. The solutions obtained at days 110, 135, and 160 are shown, respectively, in Fig S2, Fig S3, and Fig S4.

The moving horizon strategy (Fig 6) relies on state estimation for values of $e(t)$ and $a(t)$. While the control policies are only updated every 25 days, the state estimates are updated daily as new measurements are made available. The values of $e(t)$ and $a(t)$ were estimated using an unscented Kalman filter for moving horizon control. The estimates over time are shown in Fig S5.

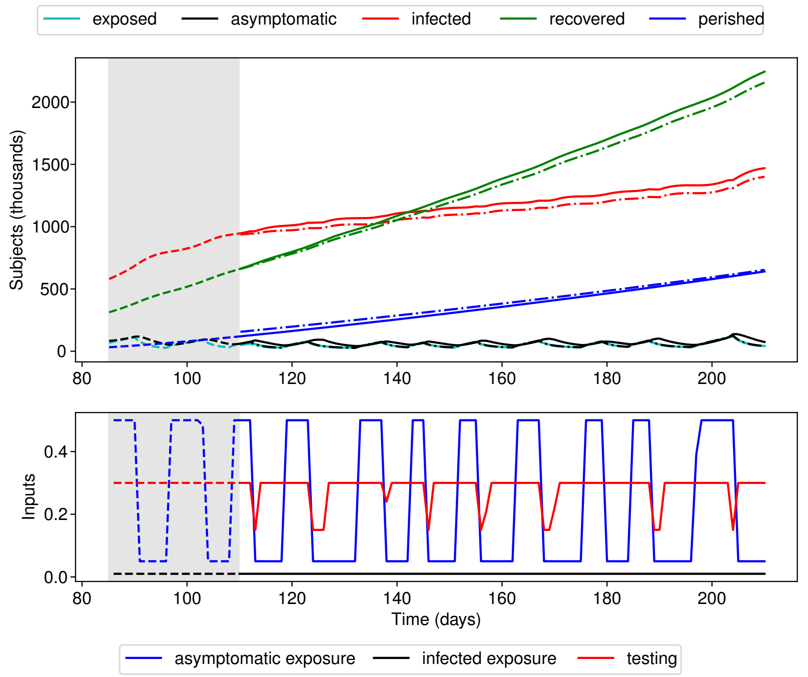


**Figure S2.** Optimal control policy found at day 110 in moving horizon scenario. Top: predicted (dash-dotted) and true (solid) population numbers. Bottom: containment and testing profiles. The shaded grey area indicates past days, which were simulated using historical inputs (not optimized).

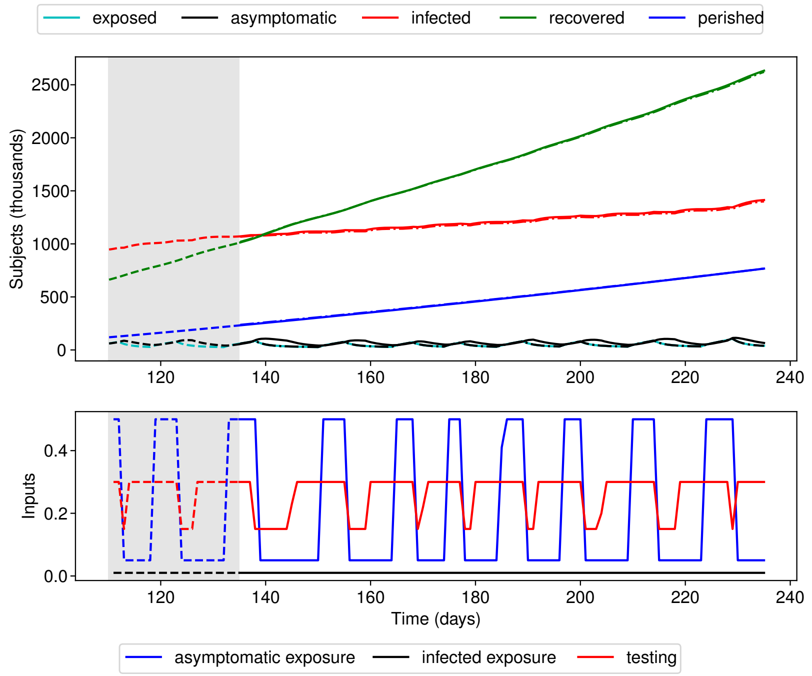


**Figure S3.** Optimal control policy found at day 135 in moving horizon scenario. Top: predicted (dash-dotted) and true (solid) population numbers. Bottom: containment and testing profiles. The shaded grey area indicates past days, which were simulated using historical inputs (not optimized).

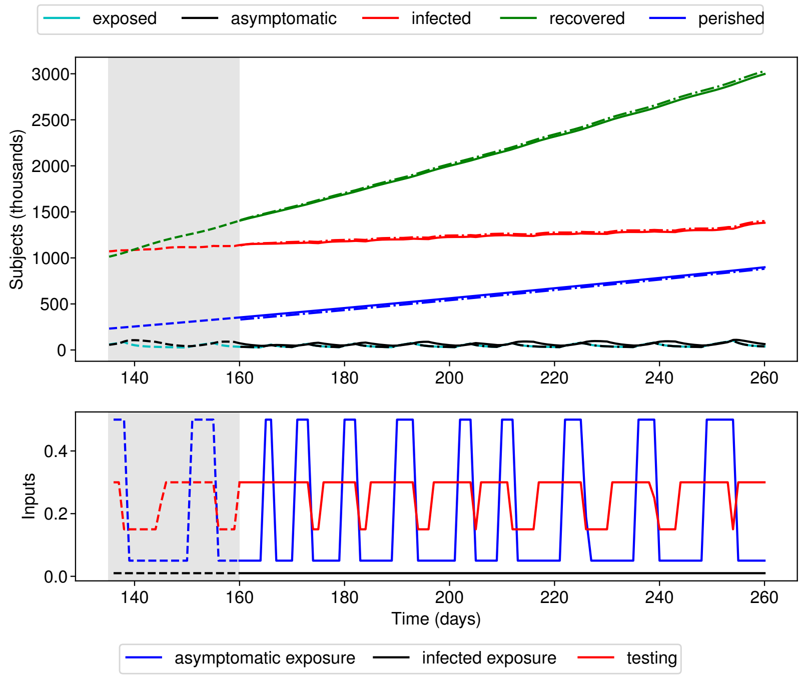


**Figure S4.** Optimal control policy found at day 160 in moving horizon scenario. Top: predicted (dash-dotted) and true (solid) population numbers. Bottom: containment and testing profiles. The shaded grey area indicates past days, which were simulated using historical inputs (not optimized).

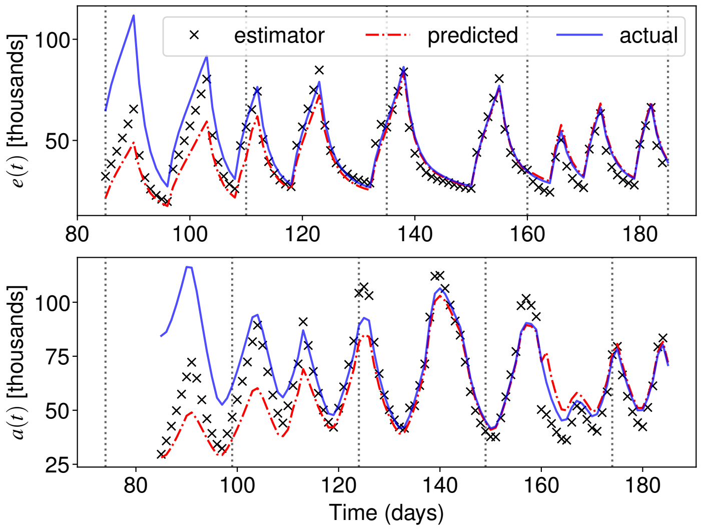


**Figure S5.** Estimation of hidden states during moving horizon control. Top: estimated, predicted (during optimization), and actual values of $e(t)$. Bottom: estimated, predicted, and actual values of $a(t)$. Re-optimization is performed every 25 days, while state estimation is performed daily.
